# Preclinical testing of the glycogen synthase kinase-3β inhibitor tideglusib for rhabdomyosarcoma

**DOI:** 10.18632/oncotarget.18520

**Published:** 2017-06-16

**Authors:** Narendra Bharathy, Matthew N. Svalina, Teagan P. Settelmeyer, Megan M. Cleary, Noah E. Berlow, Susan D. Airhart, Sunny Xiang, James Keck, James B. Hayden, Jack F. Shern, Atiya Mansoor, Melvin Lathara, Ganapati Srinivasa, David M. Langenau, Charles Keller

**Affiliations:** ^1^ Children's Cancer Therapy Development Institute, Beaverton, OR 97005, USA; ^2^ The Jackson Laboratory, Sacramento, CA 95838, USA; ^3^ Department of Orthopedics and Rehabilitation, Oregon Health & Science University, Portland, OR 97239, USA; ^4^ Genetics Branch, Oncogenomics Section, Center for Cancer Research, National Institutes of Health, Bethesda, MD 20892, USA; ^5^ Pediatric Oncology Branch, Center for Cancer Research, National Institutes of Health, Bethesda, MD 20892, USA; ^6^ Department of Pathology, Oregon Health & Science University, Portland, OR 97239, USA; ^7^ Omics Data Automation, Beaverton, OR 97005, USA; ^8^ Molecular Pathology, Cancer Center, and Regenerative Medicine, Massachusetts General Hospital, Boston, MA 02114, USA; ^9^ Harvard Stem Cell Institute, Cambridge, MA 02129, USA

**Keywords:** rhabdomyosarcoma, preclinical testing, patient-derived xenograft, GSK3β, myodifferentiation

## Abstract

Rhabdomyosarcoma (RMS) is the most common childhood soft tissue sarcoma. RMS often arise from myogenic precursors and displays a poorly differentiated skeletal muscle phenotype most closely resembling regenerating muscle. GSK3β is a ubiquitously expressed serine-threonine kinase capable of repressing the terminal myogenic differentiation program in cardiac and skeletal muscle. Recent unbiased chemical screening efforts have prioritized GSK3β inhibitors as inducers of myodifferentiation in RMS, suggesting efficacy as single agents in suppressing growth and promoting self-renewal in zebrafish transgenic embryonal RMS (eRMS) models *in vivo*. In this study, we tested the irreversible GSK3β-inhibitor, tideglusib for *in vivo* efficacy in patient-derived xenograft models of both alveolar rhabdomyosarcoma (aRMS) and eRMS. Tideglusib had effective on-target pharmacodynamic efficacy, but as a single agent had no effect on tumor progression or myodifferentiation. These results suggest that as monotherapy, GSK3β inhibitors may not be a viable treatment for aRMS or eRMS.

## INTRODUCTION

Rhabdomyosarcoma (RMS) is the most common childhood soft tissue sarcoma and is broadly classified into two histologic subtypes: alveolar rhabdomyosarcoma (aRMS) and embryonal rhabdomyosarcoma (eRMS). aRMS is a highly aggressive tumor is characterized by the pathogenomic t(1:13) or t(2:13) translocation resulting in the chimeric gene-fusion product PAX3:FOXO1 or PAX7:FOXO1, respectively [1]. eRMS is often defined as a *RAS* driven tumor [2–4]. RMS has been shown experimentally to have a myogenic cell-of-origin in some model systems and displays a poorly differentiated phenotype with gene expression profiles similar to fetal or regenerating muscle [5, 6]. Despite the expression of myogenic differentiation-specific transcription factors MYOD1 and myogenin, RMS fails to terminally differentiate [7].

Glycogen synthase kinase 3β (GSK3β) is a ubiquitously expressed serine-threonine kinase involved in the suppression of skeletal muscle myogenesis and cardiomyocyte hypertrophy via repression of MEF2 transcriptional activity and p38/MAPK signaling [8]. In myoblasts, inhibition of GSK3β induces muscle differentiation [9, 10]; thus, pharmacologic inhibition of GSK3β has been suggested to be a possible therapeutic avenue towards myodifferentiation in RMS [11].

To this end, recent studies have explored molecularly-targeted therapies that overcome the impaired differentiation in rhabdomyosarcoma [12–15]. A report from our group using unbiased chemical screens prioritized GSK3β inhibitors as inducers of myogenic differentiation in eRMS [11]. In this study, we investigated the expression levels of GSK3α and GSK3β at the mRNA and protein level in RMS patient samples, RMS cell lines, and normal samples. In addition, we examined the *in vivo* effect of pharmacologic GSK3β inhibition in aRMS and eRMS.

RNA-Seq data revealed significant differences in the expression of GSK3α/β (and its splice variants) in aRMS and eRMS. We then tested the preclinical efficacy of tideglusib, an irreversible inhibitor of GSK3β [16] in patient-derived xenograft (PDX) models of eRMS and aRMS. *In vivo*, tideglusib had no effect on tumor progression or myodifferentiation, although pharmacodynamic examination showed efficient reduction in GSK3β mediated phosphorylation of β-catenin at Ser^33/37^ and Thr^41^ and consequent stabilization of β-catenin [17].

## RESULTS

### Comparison of GSK3α and GSK3β expression in normal muscle and RMS cell lines and patient samples

**Figure 1:**
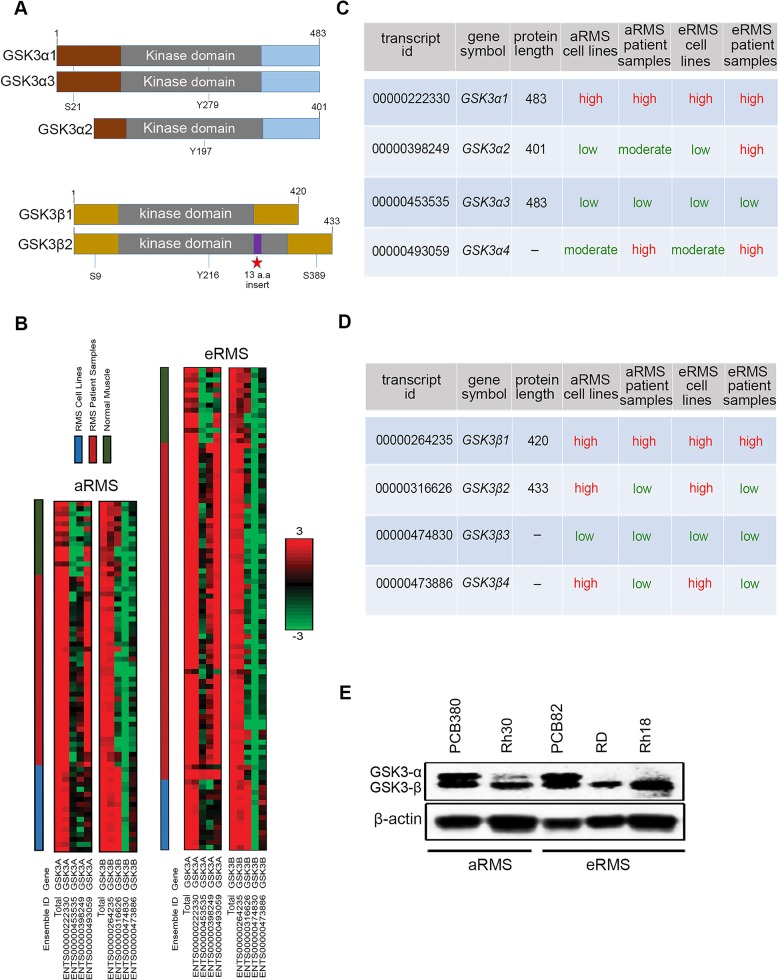
*GSK3α* and *GSK3β* expression in RMS cell lines, patient samples, and normal muscle **(A)** Schematic representation of full length GSK3α1/α2/α3 and GSK3β1/β2 showing their catalytic domain (kinase), sites of serine (S) and tyrosine (Y) phosphorylation. **(B)** RNA sequencing was performed on 31 RMS cell lines, 105 RMS patient samples, and 19 normal muscle tissue samples and the resulting Log2-scaled RPKM values for 4 isoforms of *GSK3α* and *GSK3β* are shown. Different sample types (RMS cell line, RMS patient sample, normal muscle) are indicated by the color-coded bars at the top of the figure. The heat scale is given on the side, ranging from green (low expression; RPPKM = −3), to black (RPKM = 0), to red (high expression; RPKM=3).**(C & D)** Table showing the different spliced variant of GSK3α and GSK3β with their respective ensemble ID, gene symbol, protein length (a.a) and their expression across, aRMS, eRMS patient samples and cell lines (color code matching heat map above). **(E)** Western blotting showing pattern of GSK3 *α*/β expression across aRMS, eRMS, or primary tissue versus cell line samples. GSK3α and GSK3β have molecular weight of 51 and 47 KDa.

GSK3 is a serine-threonine kinase that exists as two isoforms encoded by two distinct genes: *GSK3α* and *GSK3β* [18]. These isoforms are structurally similar (Figure 1A) but functionally different and exhibit distinct phenotypes [18]. GSK3α/*β* contain a protein kinase domain with phosphorylation of Tyr (Y^279^/^216^) facilitating its catalytic activity and phosphorylation of Ser^21/9^ inhibiting its own activity [19, 20] (Figure 1A). Although GSK3α exists as GSK3α1/α2/α3/α4, these variants are rarely specified in literature. *GSK3α1/α3* contains an open reading of 483 amino acids each encoding a protein of 51 KDa. *GSK3α2* contains an open reading of 401 amino acids encoding a protein of 45 KDa which has not been studied in detail. *GSK3α4* does not form protein [http://www.ensembl.org/Homo_sapiens/Gene/Summary?db=core;g=ENSG00000105723;r=19:42230186-42242625].

Similarly, GSK3β exist as spliced variants GSK3β1/β2/β3/β4 (Figure 1A). GSK3β1 is a predominantly expressed spliced variant with GSK3β2 generally accounting for only 15% of total expression [19]. GSK3β3/β4 does not form protein [http://uswest.ensembl.org/Homo_sapiens/Gene/Summary?db=core;g=ENSG00000082701;r=3:119821323-120094417]. GSK3β2 has 13 additional amino acids inserted in the catalytic (kinase) domain [19] (Figure 1A); however, both GSK3β1 and GSK3β2 have similar phosphorylation patterns at the regulatory sites Ser^9^ and Tyr^216^. GSK3β1/β2 exhibits preferential activity towards specific substrates; for example, GSK3β2 has reduced kinase activity towards the microtubule associated protein tau (Ser^396^), phospho-glycogen synthase 2 peptide, CRMP2 (Thr^509/514^), CRMP4 (Thr^509^) and Inhibitor-2 (Thr^72^) compared to GSK3β1 [20–21].

To begin the preclinical validation of further preclinical studies of GSK3β inhibition in RMS, we investigated the incidence of GSK3α and GSK3β expression in RMS. From a published RNA- Seq data [22], we examined GSK3α/β expression in 31 RMS cell lines (17 aRMS, 14 eRMS), 105 RMS patient samples (38 aRMS, 67 eRMS) and 19 normal muscle tissue samples. RMS sample and normal sample expression data are presented as a heatmap partitioned into disease subtype and splice variants in Figure 1B. Overall, RNA expression of GSK3α and GSK3β statistically differs amongst sample type (RMS patient samples, RMS cell lines, normal muscle samples) and disease type (aRMS, eRMS). GSK3α1 and GSK3β1 both had high expression across aRMS/eRMS patient samples, cell lines and normal muscle (Figure 1B & 1C). GSK3α2 is expressed at increased level by patient samples compared to cell lines and normal muscle, whereas GSKα3 is expressed at lower levels across patient samples and cell lines (Figure 1B & 1C). GSK3α4, which has no protein, is expressed at the RNA level in patient samples. Interestingly, GSK3α2 has no conserved serine amino acid at position twenty one unlike GSK3α1/α3. Phosphorylation of Ser^21^ inhibit GSK α1/α3 activity, raising the question whether GSK3α2 expression in aRMS and eRMS patients could be related to oncogenesis or progression. Among GSK3β, GSK3β2 is expressed at high levels in cell lines and low in patient samples. Similarly, GSK3β4 which has no protein is expressed at high levels in cell lines but at low levels in patient samples. The GSK3β3 splice variant, which has no protein, was expressed at low levels across aRMS/eRMS patient samples and cell lines (Figure 1B & 1D). Overall, GSK3α1 and GSK3β1 that are expressed most consistently at high levels across cell lines/patient samples (Figure 1B & 1C & 1D).

To compare co-expression of GSK3α and GSK3β between RMS patient samples, cell lines, and normal muscle samples for aRMS and eRMS, we measured correlation coefficients of GSK3α and GSK3β expression between samples (Supplementary Figure 1). The co-expression of GSK3α and GSK3β is statistically different (*i.e*., unlinked) amongst all sample types in aRMS (cell lines vs. patient tumors p = 1.38e^−17^, cell lines vs. normal samples p = 2.05e^−9^, patient tumors vs. normal samples p = 4.04e^−15^) and eRMS (cell lines vs. patient tumors p = 2.63e^−20^, cell lines vs. normal samples p = 1.02e^−5^, patient tumors vs. normal samples p = 4.3e^−17^) per Hotelling's T^2^ test. MANOVA statistical analysis revealed GSK3α and GSK3β expression is uncorrelated amongst individual samples and across sample types (GSK3α samples p = 2.5e^−8^, type p = 2.2e^−7^, GSK3β samples p = 1.7e^−13^, type p = 2.1e^−14^) (Figure 1B, Supplementary Figure 1).

To assess if GSK3α/β expression is correlated with clinical outcome, we surveyed a publicly available clinical dataset [23] to analyze the expression of each gene in two patient segments (fusion-positive RMS and fusion-negative RMS) with the corresponding survival of the patient segments (Supplementary Figure 2, Supplementary Table 1). GSK3α/β expression was generated using Affymetrix U95 Gene Chip microarrays, and thus is a unit less measure of gene expression. Only in fusion-positive RMS, GSK expression correlated with statistically significant difference in patient survival, with low versus high GSK3β expression (threshold = 170) showing statistical significance (p = 0.04) in patient outcome across 23 patients (Supplementary Figure 2D). Higher expression of GSK3β correlated with improved outcome in the aRMS patients.

To identify a representative xenograft model system, we performed western blot analysis of expression of GSK3α/β in aRMS/eRMS cell lines and PDX tumor cultures (Figure 1E). High GSK3α/β co-expression was seen in aRMS primary tumor culture PCB380 and eRMS primary tumor culture PCB82 in comparison to aRMS cell lines Rh30 and eRMS cell lines RD and Rh18. RNA-Seq data (GEO Accession number GSE100427) revealed higher expression of GSK3α1/β1 compared to other splice variants in PCB380/82 (Supplementary Tables 2 & 3 & 4 & 5). Thus, PDX models PCB380 and PCB82 were selected as model systems for further studies.

### Tideglusib shows pharmacological efficacy *in vitro*

**Figure 2:**
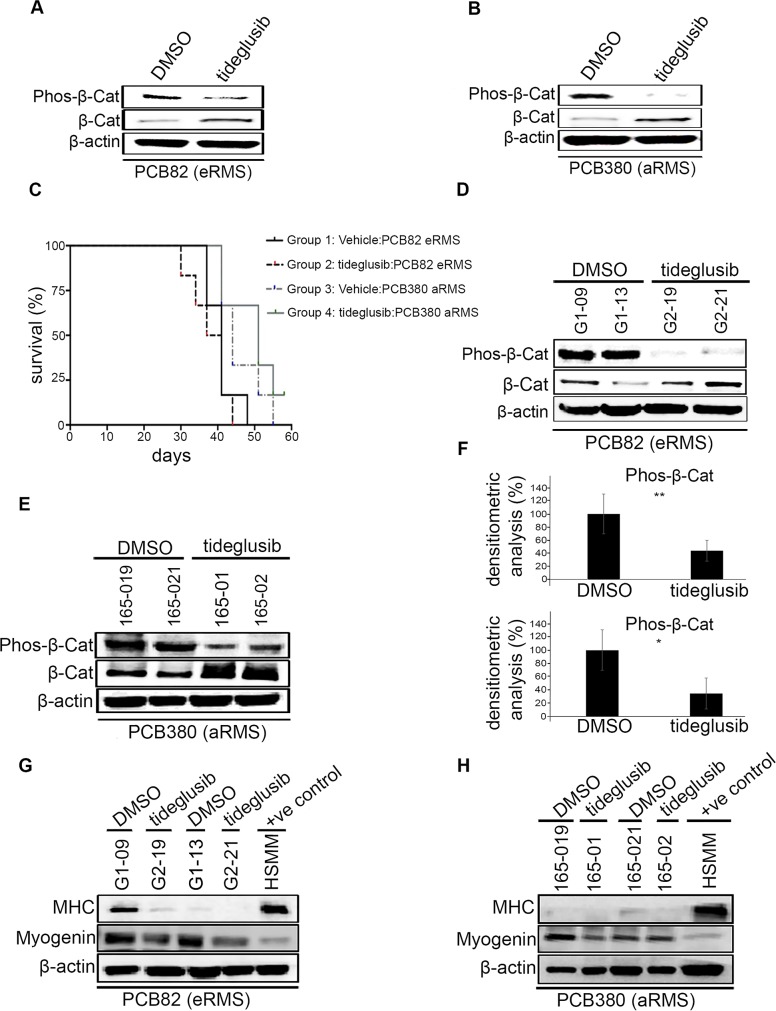
Effects of tideglusib on tumor growth, myodifferentiation *in vivo* **(A & B)** Western blotting of vehicle and tideglusib treated human PDX derived primary culture (PCB82 and PCB380) for detection of GSK-3β mediated phosphorylation of β-catenin which showed reduction and an increase in total β-catenin. **(C)** Kaplan-Meier curve showing eRMS (PCB82) and aRMS mice (PCB380) treated with 200 mg/kg tideglusib via oral gavage daily experienced no effect on survival. **(D & E)** Western blotting of vehicle and tideglusib treated human PDX tumors (PCB82 and PCB380) for detection of GSK3β mediated phosphorylation of β-catenin which showed reduction and an increase in total β-catenin. **(F)** Densitometric analysis shows the reduction in phos-β-catenin upon tideglusib treatment in eRMS (PCB82) (upper panel) and aRMS (PCB380) (lower panel) to be statistically significant (*p>0.05; **p>0.001). Error bars represent mean ± S.D. **(G & H)** Western blotting of vehicle and tideglusib treated human PDX derived primary culture (PCB82 and PCB380) for detection of myogenin and myosin heavy chain (MHC). Differentiated HSMM used as a positive control.

To test the pharmacological activity of tideglusib against GSK3β, PDX derived cell cultures PCB82 (eRMS) and PCB380 (aRMS) were treated with tideglusib at its previously reported enzyme IC50 of 60 nM, which has been shown to bring irreversible inhibition of kinase activity of GSK3β (in cell free assay) [16]. After twenty minutes, cells were harvested and analyzed for the GSK3β mediated phosphorylation status of β–catenin, which was reduced substantially upon treatment. A corresponding increase in total levels of β–catenin was also observed which indicated the efficacy of tideglusib in blocking GSK3β activity (17) (Figure 2A & 2B).

### Tideglusib shows no *in vivo* effect on survival or myodifferentiation

We performed preclinical testing of tideglusib in PDX mouse models of aRMS (PCB380) and eRMS (PCB82). The maximum tolerated dose (MTD) of tideglusib was determined experimentally and found to be 200 mg/kg (data not shown). No significant toxicity (weight loss, activity change) was observed at this dose.

Tumor-bearing mice were treated with 200 mg/kg of tideglusib daily by oral gavage. Kaplan-Meier survival analysis showed no significant differences in survival between Group 1 and Group 2 (PCB82 eRMS treated with vehicle vs tideglusib, p = 0.972) or Group 3 and Group 4 (PCB380 aRMS treated with vehicle vs tideglusib, p = 0.612) (Figure 2C). Pharmacodymanic analysis showed significant reduction in the GSK3β mediated phosphorylation of β-catenin and an increase in total β-catenin in tumor lysates (Figure 2D & 2E) in both eRMS (p = 0.038) (Figure 2F; upper panel) and aRMS model (p = 0.024) (Figure 2F; lower panel) demonstrating that tideglusib treatment inhibits catalytic activity of GSK3β but does not improve survival.

We also examined the effect of tideglusib on myodifferentiation. Immunohistochemistry on treat-ment and control groups from both aRMS and eRMS PDX model did not exhibit any rhabdomyoblasts (Supplementary Figure 3 & Supplementary Table 6). Western blot analysis of tideglusib-treated aRMS and eRMS PDX tumor protein lysates showed no induction of myodifferentiation (via myosin heavy chain expression) in treated versus control groups. Differentiated human skeletal myoblasts (HSMM) were used as a positive control (Figure 2G & 2H). Following tideglusib treatment, myogenin was downregulated in eRMS (Figure 2G), and aRMS (Figure 2H). Overall, tideglusib had no effect on survival or myodifferentiation.

## DISCUSSION

To our knowledge this is the first study 1) to analyze the expression of GSK3α/β and its splice variants across a broad collection of RMS cell lines and patient samples in comparison to normal muscle samples, and 2) to examine pharmacological inhibition of GSK3β in PDX RMS models.

Tideglusib, an irreversible inhibitor of GSK3β, was tested for preclinical efficacy against RMS PDX models. Tideglusib displayed pharmacokinetic potency in reducing GSK3β-mediated phosphorylation of phospho-β-catenin and consequent increase in total β-catenin *in vitro* for primary tumor cultures with demonstrated over-expression of GSK3α and GSK3β. Despite this on-target efficacy, tideglusib tested at the highest dosage (200 mg/kg) had no effect on *in vivo* tumor growth or myodifferentiation in PDX models of aRMS or eRMS. Unexpectedly, tideglusib treatment showed reduction in myogenin in aRMS/eRMS PDX tumor. This is surprising as recent study shows that myogenin function in aRMS is disrupted by sustained GSK3β kinase activity contributing to the undifferentiated, proliferative phenotype [13]. However, the difference in observation could be attributed to the fact that this study is limited to one aRMS cell line, Rh30. Our studies of GSK3α/β isoform expression suggest that cell lines have a skewed isoform and splice variant expression compared to primary tumor tissue, which is important to take into account. Our findings also suggests that GSK3β is not the optimal therapeutic target for single-agent treatment of aRMS/eRMS. Yet while tideglusib may be of limited value as a monotherapy in RMS, future studies could explore combination therapies with chemotherapy agents. Furthermore, since GSK3 is regulated by multiple pathways, new roles of GSK3α and GSK3β, under non-homeostatic ‘stress’ conditions may be uncovered.

## MATERIALS AND METHODS

### Cell culture

aRMS cell line Rh30, eRMS cell lines RD and Rh18, and PDX cultures PCB380 and PCB82 were cultured in growth medium (GM) RPMI 1640 (11875-093; Thermo Fisher Scientific, Waltham, MA, USA) supplemented with 10% fetal bovine Serum (FBS) (26140079; Thermo Fisher Scientific) and 1% penicillin/Streptomycin (15140-122; Thermo Fisher Scientific). Primary human skeletal myoblasts (HSMM) (CC-2580; Lonza Inc, Allendale, NJ, USA) were cultured in GM (SKBM-2 CC-3244; Lonza Inc) supplemented with 10% FBS and 1% penicillin/streptomycin. HSMM was differentiated until day 4 in differentiation medium DM: F-12 (11320033; Thermo Fisher Scientific). All cells were incubated at 37°c and 5% CO_2_. aRMS and eRMS cell lines were authenticated by short tandem repeat (STR) analysis (Biosynthesis, Lewisville, TX, USA).

### Tideglusib cell treatments

Tideglusib, an irreversible inhibitor of GSK3β was purchased from SelleckChem (S2823, Houston, TX, USA). aRMS and eRMS PDX primary tumor cell cultures were treated with tideglusib at its IC50 for GSK3β (60 nM) [16].

### Patient-derived xenograft models

RMS samples were collected from patients undergoing planned surgical resection or research-autopsy enrolled in the bio-banking and model development program Childhood Cancer Registry for Familial and Sporadic Tumors (CuRe-FAST). All patients provided informed consent; patient data and clinical and pathologic findings are maintained in a de-identified database. All aspects of the study were reviewed and approved by the Oregon Health & Science University Institutional Review Board and the Children's Cancer Therapy Development Institute Institutional Review Board. The eRMS PDX PCB82 (TM00360) was recently described [24], and the Pax3:Foxo1+ aRMS PDX model PCB380 (TM01165) was similarly established from an untreated, surgically-resected soleus tumor from a 2-year-old female. To establish each PDX model, NSG (NOD.Cg-*Prkdc^scid^ IL2rg^tm1Wjl/SzJ^*) mice were obtained by The Jackson Laboratory. Tumor fragments were obtained from the aforementioned patients and implanted into the rear flanks of recipient female NSG (JAX # 5557) mice using a trochar. Once tumors reached 1cc, they were collected and fragmented for serial transplantation in NSG mice to create low-passage cohorts for future studies. All studies were done with the approval of The Jackson Laboratory IACUC. For tissue culture work, PCB380 and PCB82 were authenticated by analysing for the expression of myogenin and lack of MHC expression.

### Generation of RNA-Seq data

RNA sequencing data has been previously described [22].

### Western blot

To prepare protein lysate, tumors and PDX cell lines PCB82 and PCB380 treated with vehicle (control) and tideglusib were lysed in radio immunoprecipitation (RIPA) buffer (89901, Thermo Fisher Scientific) containing both protease and phosphatase inhibitors (Sigma Aldrich, St. Louis, MO). Lysates were homogenized and clarified by centrifugation at 14,000 rpm for 10 minutes. Thirty μg of protein were electrophoresed in 7.5-10% mini protean polyacrylamide gel (4561024, Bio-Rad, Hercules, CA, USA) transferred to PVDF membranes (1620255, Bio-Rad) for immunoblot analysis with anti-GSK3α/β (sc-7291, Santa Cruz Biotechnology, Dallas, TX, USA) (the monoclonal antibody used is raised against the amino acid 1-420 representing the full length GSK3β), anti-Phos-β-Catenin (9561, Cell Signaling, Danvers, MA, USA), anti-β-Catenin (9562, Cell Signaling), anti-MHC (MAB4470, R&D systems, Minneapolis, MN, USA), anti-myogenin (sc-576, Santa Cruz Biotechnology), anti-β-actin (ab8227, Abcam, San Francisco, CA, USA). Blots were developed using FluorChem Q system (92-14095-00, protein simple, San Jose, CA, USA).

### Xenograft studies

Tideglusib was dissolved in 30% PEG400/0.5% Tween80/5% propylene glycol, 30 mg/mL and formulated for *in vivo* testing in 26% PEG400 (Polyethylene Glycol 400), 15% Cremophor EL. The MTD of tideglusib was determined experimentally. Mice (n=3 per arm) were randomized to 100, 150, or 200 mg/kg treatment arms. Mice were dosed by oral gavage daily for 21 days. Clinical observations of general behavior, mobility, and weight loss were performed daily. MTD was defined as the dosage that causes a mean weight loss of >20% as a group or a body condition of <2 for one or more animals in a group. For PDX preclinical efficacy studies, forty-eight 5-6 week old NSG mice were obtained from The Jackson Laboratories. Twenty-four mice were trochar implanted with a passage 1-3 PDX fragment in the right hind flank to generate study mice. Mice were monitored twice weekly for clinical observations and body weight. Tumor-bearing mice were randomized into treatment cohorts of n=6 mice per group so that the mean cohort tumor volume was 200-300 cc. Mice were treated with vehicle or tideglusib administered by oral gavage at a dose of 200 mg/kg daily until tumors reached 2000 cc or the condition of the mice precluded ongoing treatment.

### Statistical analysis

The tumor endpoint volumes for time-to-event (TTE) analysis were set at 1500 mm^3^ and 2000 mm^3^, in the tideglusib study. TTE was defined in days by selecting the day in which the tumor volume equaled or surpassed 1500 mm^3^ and 2000 mm^3^. Animals that did not reach endpoint volume were assigned a TTE of 58 days. Comparisons were analyzed by One-way Anova with Bonferroni's post-test. The Kaplan-Meier survival plot represents the percentage of animals surviving at different time points during the study. These percentages were generated from the TTE data using GraphPad Prism 6.0 software. Survival curve comparisons were analyzed by the Mantel-Cox and Gehan-Breslow-Wilcox tests (95% CI) through Graph Pad Prism software. For densitometric analysis, significance was determined by a one-tailed Student's t test and *p* values of <0.05 were considered to be statistically significant. Statistical significance were set at * P < 0.05 and **P < 0.01. Error bars indicate mean ± standard deviation (SD).

## SUPPLEMENTARY MATERIALS FIGURES AND TABLES


