# Supplementary material for: Preclinical testing of the glycogen synthase kinase-3β inhibitor tideglusib for rhabdomyosarcoma
Source: Oncotarget. 2017 Jun 16;8(38):62976–83. doi: 10.18632/oncotarget.18520 (PMC5609896; doi:10.18632/oncotarget.18520)
Supplement: Supplementary file 1 [file oncotarget-08-62976-s001.pdf]

Preclinical testing of the glycogen synthase kinase-3β inhibitor tideglusib for rhabdomyosarcoma

SUPPLEMENTARY MATERIALS

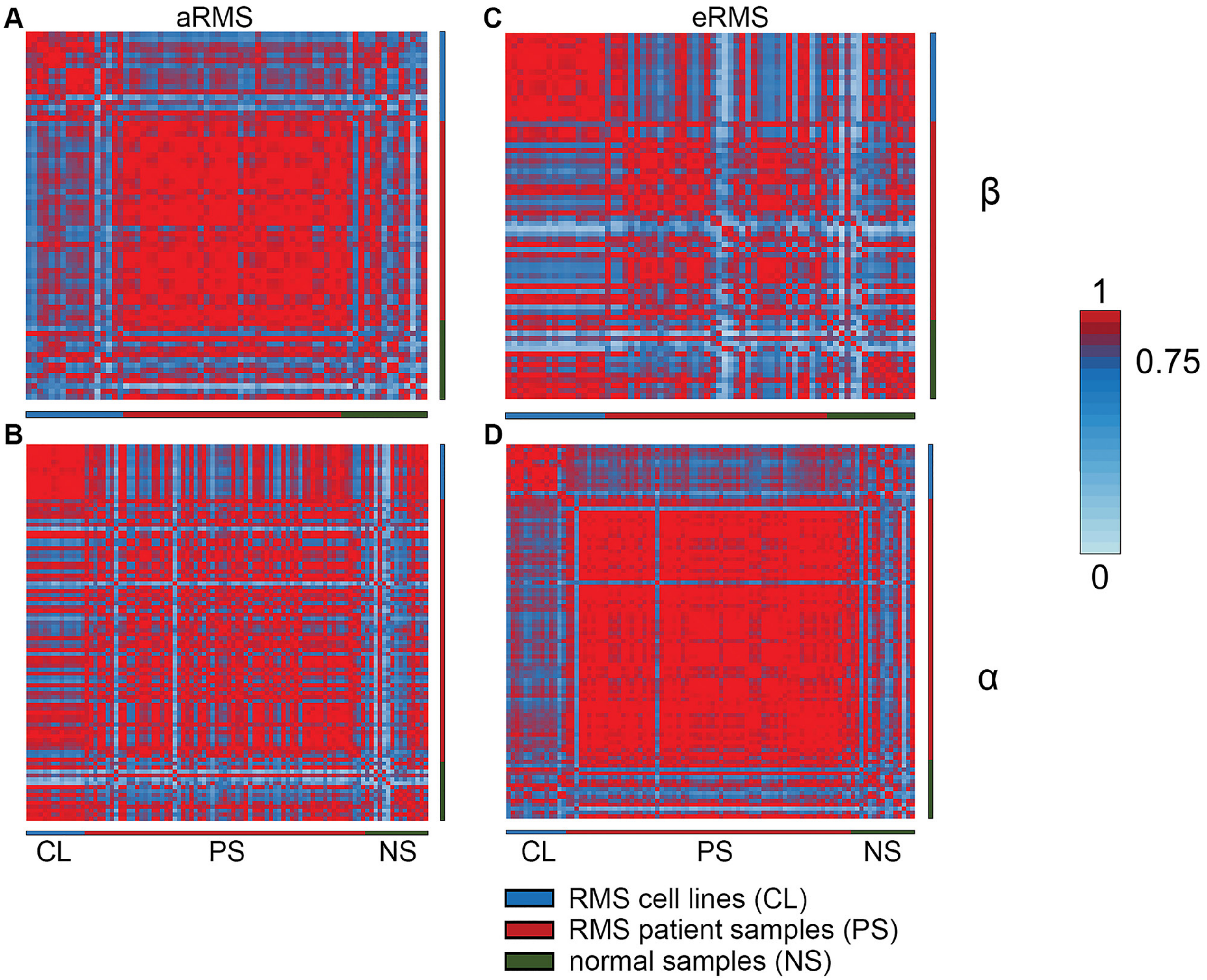

Supplementary Figure 1: Pearson correlation coefficients of GSK3α and GSK3β isoform expression of all samples. Breakdown between sample types (RMS cell line, RMS patient sample, normal muscle) is indicated by thin black lines and by the color-coded bars to the right and bottom of each figure. The heat scale is given on the side, ranging from light blue (no correlation), to dark blue (some correlation), to red (complete correlation). **(A)** Correlation coefficients of 4 GSK3β isoforms in all aRMS samples. **(B)** Correlation coefficients of 4 GSK3α isoforms in all aRMS samples. **(C)** Correlation coefficients of 4 GSK3β isoforms in all eRMS samples. **(D)** Correlation coefficients of 4 GSK3α isoforms in all eRMS samples.

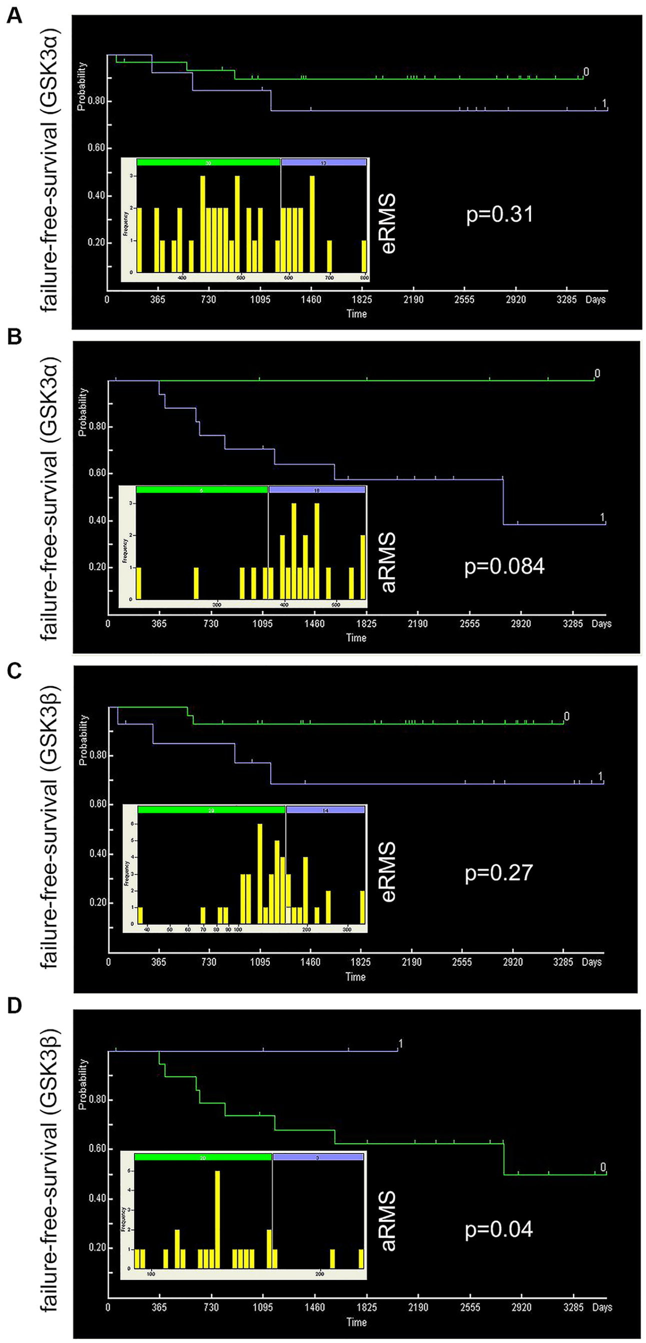

Supplementary Figure 2: Kaplan-Meier survival curves of fusion-positive and fusion-negative human RMS cases stratified by binary GSK3α and GSK3β gene expression. **(A)** Kaplan-Meier survival curves of fusion-negative RMS segmented by high versus low (threshold = 580) GSK3α expression. **(B)** Kaplan-Meier survival curves of fusion-positive RMS segmented by high versus low (threshold = 375) GSK3α expression. **(C)** Kaplan-Meier survival curves of fusion-negative RMS segmented by high versus low (threshold = 167) GSK3β expression. **(D)** Kaplan-Meier survival curves of fusion-positive RMS segmented by high versus low (threshold = 170) GSK3β expression. Higher expression of GSK3β correlated with improved outcome in the aRMS patients.

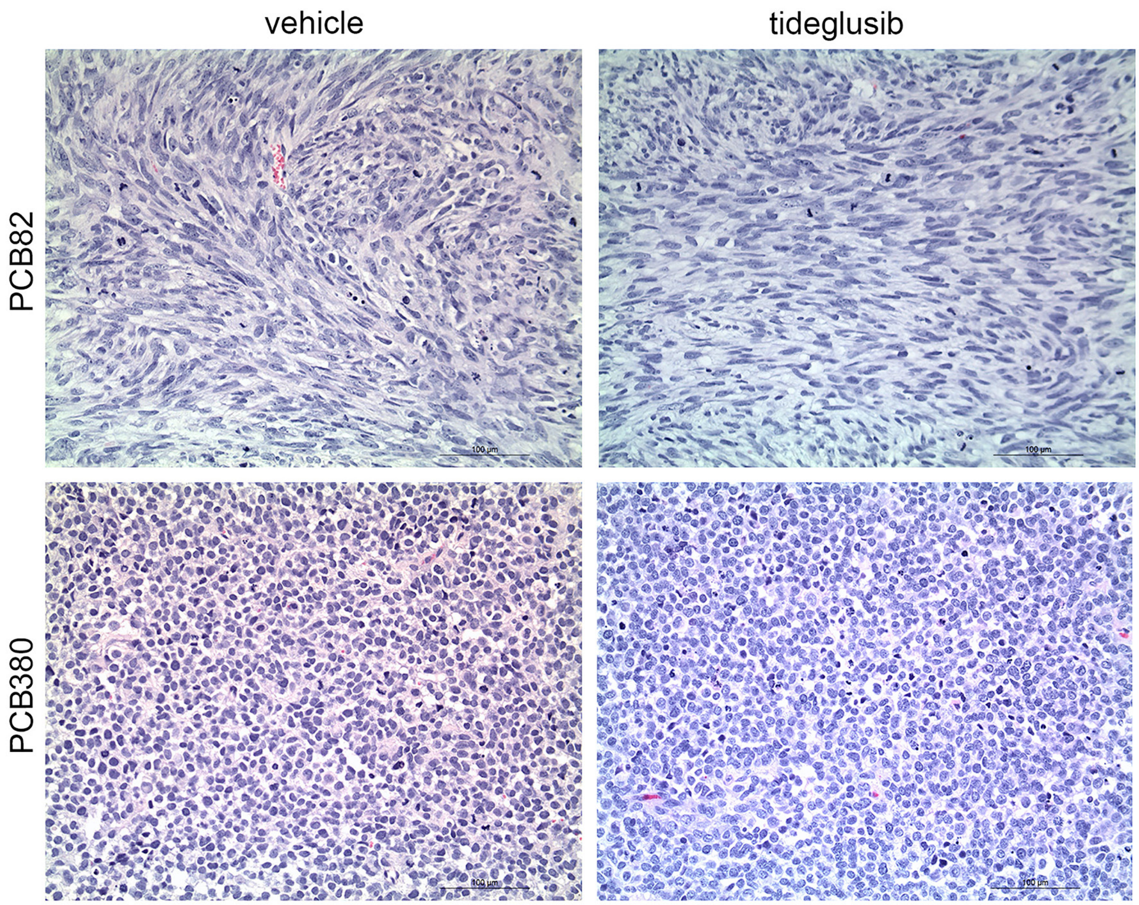

Supplementary Figure 3: Representative histology of tumors from eRMS and aRMS mice treated with vehicle and tideglusib. Samples were collected from mice upon necropsy at study completion. Tumors were stained by hematoxylin and eosin and scored by a pathologist. No rhabdomyoblasts visible in tideglusib treated cells. Scale bar = 100 µm.

Supplementary Table 1: GSKα/β expression and its correlation with clinical outcome

Gene	Disease subtype	Threshold	p-value
GSK3α	Fusion positive	375	0.084
GSK3α	Fusion negative	580	0.31
GSK3β	Fusion positive	170	0.04
GSK3β	Fusion negative	167	0.27

Tabulation of the expression of each gene in two patient segments (fusion-positive RMS and fusion-negative RMS) with the corresponding survival of the patient segments.

Supplementary Table 2: *GSK3α* expression in PCB380

Transcript ID	Gene ID	TPM	FPKM
ENST00000222330_GSK3A-001	ENSG00000105723_GSK3A	30.84	24.02
ENST00000398249_GSK3A-002	ENSG00000105723_GSK3A	1.44	1.12
ENST00000453535_GSK3A-003	ENSG00000105723_GSK3A	2.26	1.76
ENST00000493059_GSK3A-004	ENSG00000105723_GSK3A	14.22	11.07

Tabulation of the pattern of expression of *GSK3α* splice variants. TPM (Transcripts per Million); FPKM (Fragments per Kiliobase Million).

Supplementary Table 3: *GSK3β* expression in PCB380

Transcript ID	Gene ID	TPM	FPKM
ENST00000264235_GSK3B-001	ENSG00000082701_GSK3B	8.56	6.66
ENST00000316626_GSK3B-002	ENSG00000082701_GSK3B	0	0
ENST00000474830_GSK3B-003	ENSG00000082701_GSK3B	0.19	0.15
ENST00000473886_GSK3B-004	ENSG00000082701_GSK3B	0.37	0.29

Tabulation of the pattern of expression of *GSK3β*splice variants. TPM (Transcripts per Million); FPKM (Fragments per Kiliobase Million).

Supplementary Table 4: *GSK3α* expression in PCB82

Transcript ID	Gene ID	TPM	FPKM
ENST00000222330_GSK3A-001	ENSG00000105723_GSK3A	30.99	23.36
ENST00000398249_GSK3A-002	ENSG00000105723_GSK3A	1.24	0.94
ENST00000453535_GSK3A-003	ENSG00000105723_GSK3A	1.2	0.91
ENST00000493059_GSK3A-004	ENSG00000105723_GSK3A	29.44	22.19

Tabulation of the pattern of expression of *GSK3α* splice variants. TPM (Transcripts per Million); FPKM (Fragments per Kiliobase Million).

Supplementary Table 5: *GSK3β* expression in PCB82

Transcript ID	Gene ID	TPM	FPKM
ENST00000264235_GSK3B-001	ENSG00000082701_GSK3B	10.03	7.56
ENST00000316626_GSK3B-002	ENSG00000082701_GSK3B	0.18	0.14
ENST00000473886_GSK3B-004	ENSG00000082701_GSK3B	1.22	0.92
ENST00000474830_GSK3B-003	ENSG00000082701_GSK3B	0	0

Tabulation of the pattern of expression of *GSK3β*splice variants. TPM (Transcripts per Million); FPKM (Fragments per Kiliobase Million).

Supplementary Table 6: Histological markers of differentiation in eRMS/aRMS PDX mice

Animal	Model	Treatment	Tumor morphology	% Rhabdomyoblasts	Lung metastases	Met with % Rhabdomyoblasts
165-001	aRMS	Tideglusib	Round cell	0	0	None
165-002	aRMS	Tideglusib	Round cell	0	0	None
165-004	aRMS	Vehicle	Round cell	0	0	None
165-016	aRMS	Vehicle	Round cell	0	0	None
165-019	aRMS	Vehicle	Round cell	0	0	None
165-021	aRMS	Vehicle	Round cell	0	0	None
G1-009	eRMS	Vehicle	Spindle cell	0	0	None
G1-013	eRMS	Vehicle	Spindle cell	0	0	None
G1-017	eRMS	Vehicle	Spindle cell	0	0	None
G2-03	eRMS	Tideglusib	Spindle cell	0	0	None
G2-019	eRMS	Tideglusib	Spindle cell	0	0	None
G2-021	eRMS	Tideglusib	Spindle cell	0	0	None

eRMS/aRMS mice received vehicle and tideglusib. After reaching study endpoints mice were euthanized and their tumors were surveyed for the percentage (%) of rhabdomyoblasts. 500 to 3,000 cells were counted under low and higher magnification on each slide. This count excluded endothelial cells and inflammatory cells.
